# Evaluating the Antioxidant, Cytoprotective and Wound-Healing-Associated Effects of *Haberlea rhodopensis* Ethanolic Extract in Human Skin Keratinocytes

**DOI:** 10.3390/ijms27104262

**Published:** 2026-05-11

**Authors:** Antreas Ermogenous, Eleni Sarigiannidou, Maria Psomiadou, Afroditi Panagiotidou, Georgia Persephoni Voulgaridou, Despoina Eugenia Kiousi, Daniela Moyankova, Dimitar Djilianov, Alex Galanis, Aglaia Pappa

**Affiliations:** 1 Department of Molecular Biology & Genetics, School of Health Sciences, Democritus University of Thrace, 68100 Alexandroupolis, Greece; aermogen@mbg.duth.gr (A.E.); helensarig@gmail.com (E.S.); mpsomiadou27@gmail.com (M.P.); afroditi.c.panagiotidou@gmail.com (A.P.); gvoulgar@med.duth.gr (G.P.V.); dkiousi@mbg.duth.gr.com (D.E.K.); agalanis@mbg.duth.gr (A.G.); 2 Laboratory of Physiology, Department of Medicine, School of Health Sciences, Democritus University of Thrace, 68100 Alexandroupolis, Greece; 3 Department of Functional Genetics, Abiotic and Biotic Stress, Agrobioinstitute, Agricultural Academy, 8 Dragan Tzankov Blvd., 1164 Sofia, Bulgaria; dmoyankova@abi.bg (D.M.); d_djilianov@abi.bg (D.D.)

**Keywords:** *Haberlea rhodopensis* extract, HaCaT keratinocytes, oxidative stress, antioxidant activity, cytoprotection, cellular defense mechanisms, redox regulation, wound healing

## Abstract

The resurrection plant *Haberlea rhodopensis* is a rare species endemic to Greece and Bulgaria, renowned for its exceptional desiccation tolerance and rich phytochemical composition. This study investigated the antioxidant, cytoprotective, and wound-healing-associated effects of *H. rhodopensis* ethanolic extract (HEE) in human keratinocytes (HaCaT cells) under oxidative and cytotoxic stress conditions. Antioxidant capacity was initially evaluated using a plasmid DNA protection assay, in which HEE attenuated oxidative DNA damage induced by a Fenton reaction system and preserved the native supercoiled structure of pUC19 plasmid DNA. Cytotoxicity screening using the sulforhodamine B (SRB) assay and real-time proliferation monitoring (HoloMonitor^®^ M4) identified 20 μg/mL as a non-toxic pre-treatment concentration (EC_10_). Under hydrogen peroxide (H_2_O_2_)-induced oxidative stress, HEE pre-treatment maintained cell viability and significantly reduced intracellular reactive oxygen species (ROS) levels, indicating a protective effect. In vitro wound-healing assays demonstrated enhanced scratch closure in keratinocyte monolayers. RT-qPCR analysis revealed modulation of antioxidant-related genes (*CAT*, *SOD1*, *HMOX1*, *NQO1*, *GPX*, *GSR*), while mRNA sequencing suggested selective stress-adaptive responses, involving extracellular matrix (ECM)-, metabolic-, and tissue-repair/aging-associated pathways. Overall, HEE exhibits antioxidant and cytoprotective effects in keratinocytes and is associated with transcriptional changes linked to cellular stress responses and wound closure. These findings support its potential relevance for dermatological, pharmaceutical, and cosmeceutical applications, while further studies are required to establish the underlying molecular mechanisms.

## 1. Introduction

*Haberlea rhodopensis*, also known as “Orpheus flower”, is a species belonging to the family *Gesneriaceae*. Native to the mountains in Bulgaria and Northern Greece, *H. rhodopensis* is considered a protective species due to its limited growth [1,2]. It belongs to the group of resurrection plants, which have developed unique adaptations to thrive in environments with erratic water availability. During desiccation events, the plant activates tightly regulated biochemical pathways that safeguard cellular integrity. Among the most critical mechanisms is the accumulation of osmoprotectants, such as raffinose, which contribute to the stabilization of cellular structures and membranes under dehydration stress [3,4]. Notwithstanding these adaptive traits and its extraordinary tolerance to abiotic stress at the cellular level, *H. rhodopensis* remains ecologically vulnerable due to its restricted distribution and specialized habitat.

In a previous study by Spyridopoulou et al., (2022), *H. rhodopensis* ethanolic extract (HEE) was chemically characterized and found to be rich in flavonoids, phenolic compounds, pigments (chlorophylls, β-carotene, lycopene), monoterpenoids, and condensed tannins, providing a well-defined profile for mechanistic studies [5]. Phytochemicals evolved in resurrection plants demonstrate diverse biological activities and may represent a valuable and underexplored source of bioactive compounds for skin protection and regeneration [6,7]. Specifically, under desiccation-related stress conditions, robust protective mechanisms are essential for preserving plant integrity during the dehydration–rehydration cycle, which imposes mechanical, structural, metabolic, and chemical stresses, ultimately resulting in cellular damage [8]. In particular, phenolic compounds act as direct antioxidants by scavenging ROS and also exert indirect antioxidant effects through the activation of endogenous protective enzymes and the regulation of relevant signaling pathways [9]. The elevated levels of phenolic acids and flavonoids in *H. rhodopensis* extracts confer antioxidant potential, enabling effective neutralization of ROS and protection of cells against oxidative damage [10]. Among the most abundant phenolic acids identified in the plant’s extracts are ferulic acid, caffeic acid, and p-coumaric acid [5]. The antioxidant properties of *H. rhodopensis* have been widely studied, particularly in the context of its ability to protect cells from oxidative stress [11,12]. Previous studies have shown that the plant’s extracts mitigate oxidative damage to DNA and cellular membranes, thereby providing effective protection against radiation-induced stress [10,13,14].

Human skin is constantly exposed to environmental stressors, including UV radiation, air pollutants, and chemical irritants, which lead to excessive ROS generation [15]. Keratinocytes, the major cell type of the epidermis, play a crucial role in maintaining skin barrier integrity and orchestrating antioxidant, regenerative, and stress-response mechanisms under oxidative conditions [16]. The immortalized human keratinocyte cell line HaCaT represents a well-established in vitro model for studying epidermal stress response, cytoprotection, and wound healing, making it particularly suitable for evaluating skin-relevant bioactivities of natural bio-compounds [13,14,17].

In the present study, we aimed to investigate the response of human HaCaT keratinocytes to oxidative stress following pre-treatment with an ethanolic extract of *H. rhodopensis* and subsequent exposure to H_2_O_2_. By integrating plasmid DNA protection assays, intracellular ROS measurements, gene expression analysis of key antioxidant pathways, wound healing assays, and transcriptomic analysis (mRNA sequencing), we sought to elucidate the cellular responses associated with skin-protective potential of this resurrection plant. Our findings indicate that *H. rhodopensis* exerts protective effects on plasmid DNA and displays in vitro cytoprotective properties by modulating the transcriptional regulation of genes involved in antioxidant defense, redox homeostasis, and the DNA damage response.

## 2. Results

### 2.1. Antioxidant Activity of HEE Assessed by a Plasmid DNA Protection Assay

**Figure 1:**
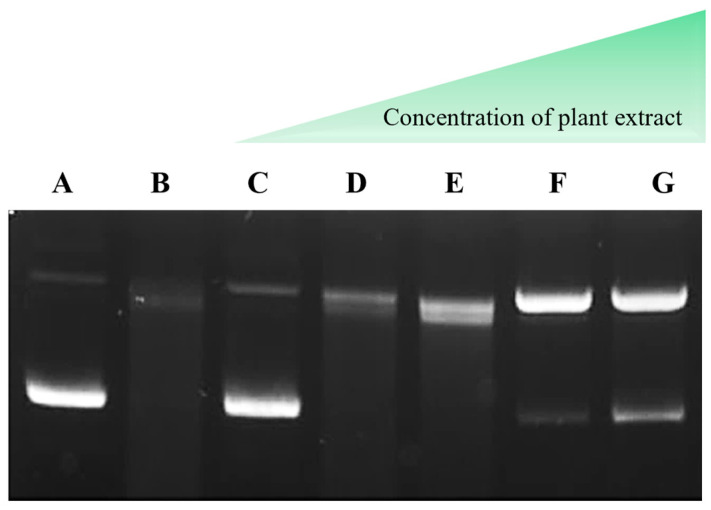
Protective effect of HEE against oxidative DNA damage induced by a Fenton reaction system (Fe^2+^ + H_2_O_2_) in pUC19 plasmid DNA. Lane A: pUC19 plasmid DNA (control); Lane B: pUC19 plasmid DNA treated with Fenton’s reagent; Lane C: pUC19 plasmid DNA incubated with HEE (2 mg/mL); Lane D: pUC19 plasmid DNA incubated with HEE (0.002 mg/mL); Lane E: pUC19 plasmid DNA incubated with HEE (0.02 mg/mL); Lane F: pUC19 plasmid treated with Fenton’s reagent in the presence of HEE (0.2 mg/mL); Lane G: pUC19 plasmid treated with Fenton’s reagent in the presence of HEE (2 mg/mL). The fast-migrating lower band corresponds to the native supercoiled form of plasmid DNA, whereas slower-migrating upper bands correspond to relaxed/open circular or linear forms resulting from oxidative damage. Images are representative of three independent experiments (n = 3).

The antioxidant capacity of HEE was evaluated using a DNA protection assay against oxidative damage induced by Fenton’s reagent. Hydroxyl radicals generated during the Fenton reaction interact with DNA bases and the sugar–phosphate backbone, leading to single- and double-strand breaks and consequent alterations in plasmid topology. pUC19 plasmid DNA was incubated with Fenton’s reagent in the presence of HEE in a concentration gradient. As shown in Figure 1, the untreated pUC19 plasmid DNA (control) migrated predominantly in the supercoiled circular form (fast-migrating lower band). Exposure to Fenton’s reagent resulted in pronounced oxidative damage, evidenced by the conversion of the supercoiled DNA into linear and relaxed/open circular forms (slower-migrating upper bands). In contrast, co-incubation with increasing concentrations of HEE attenuated Fenton-induced DNA damage, as reflected by partial preservation of the supercoiled form and a reduction in the formation of linear DNA, rather than complete restoration of the native plasmid profile. The prominent upper band observed at higher extract concentrations is consistent with relaxed/open circular DNA, representing partially protected intermediates that arise under oxidative conditions. This protective effect was particularly evident at an extract concentration of 0.2 mg/mL, indicating a concentration-dependent antioxidant activity of HEE.

### 2.2. Cytotoxicity Effect of Graded Concentrations of HEE in HaCaT Cells

**Figure 2:**
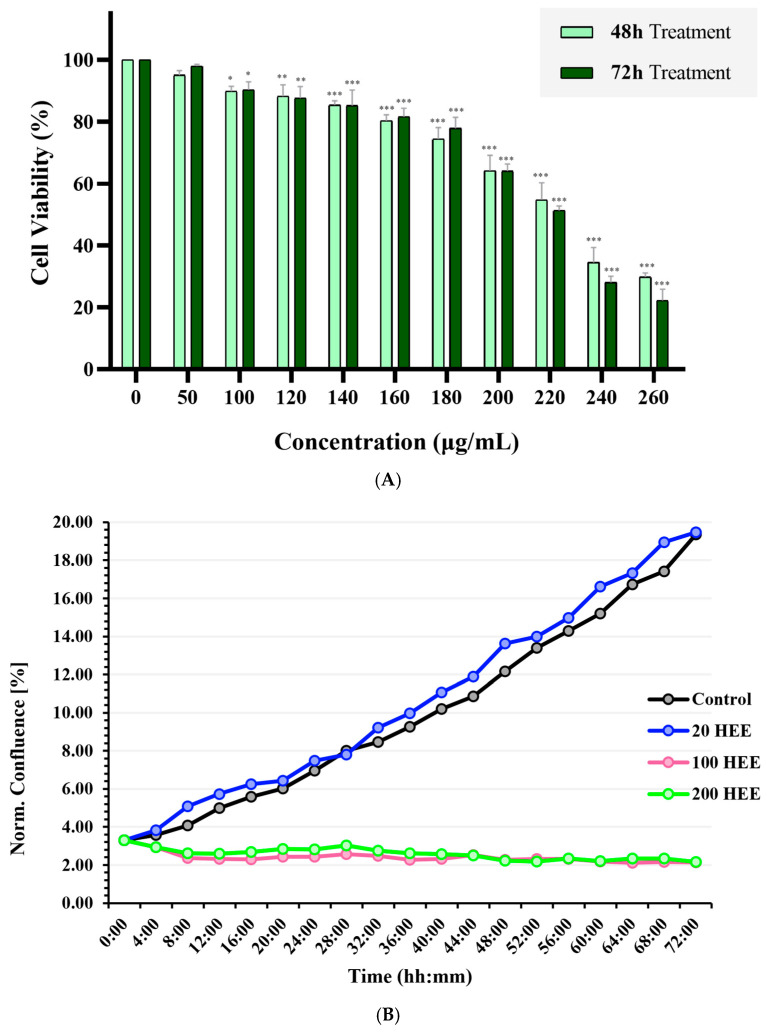
Cytotoxic effects of graded concentrations of HEE in HaCaT cells. (**A**) Cytotoxicity of graded concentrations of HEE in HaCaT cells after 48 h and 72 h of exposure, as determined by the SRB assay. Data are presented as mean ± SD (n = 3) from three independent experiments. Asterisks denote statistical significance of *p*-value (* *p* < 0.05, ** *p* < 0.01, *** *p* < 0.001) vs. control (untreated cells). (**B**) Real-time monitoring of cytotoxic effects of graded HEE concentrations in HaCaT cells using the HoloMonitor^®^ M4 Live Cell Imaging System at 4 h intervals over 72 h. Normalized confluence (%) averaged over time was quantified from ≥3 distinct frame positions per well using HStudio™ software version 4.0.2. Data shown are representative of three independent experiments (n = 3).

The cytotoxic effects of HEE in HaCaT cells were evaluated using the SRB assay following 48 h and 72 h of exposure. As shown in Figure 2A, low concentrations of HEE (≤160 μg/mL) induced only minor reductions in cell viability, with values remaining above ~80–90% compared with untreated controls. A clear dose-dependent cytotoxic response was observed at concentrations ≥ 160 μg/mL, with progressively reduced viability at both time points. Exposure to higher concentrations (≥220 μg/mL) resulted in pronounced cytotoxicity, with cell viability decreasing by more than 50% after 48 h and approximately 65% after 72 h. Overall, HEE exhibited a concentration-dependent inhibitory effect in HaCaT cell growth, with greater cytotoxicity observed following prolonged exposure.

The effects of HEE on HaCaT cell proliferation were also evaluated by real-time monitoring using the HoloMonitor^®^ M4 system over a 72 h period. As shown in Figure 2B, cells treated with a low concentration of HEE (20 μg/mL) exhibited proliferation rates comparable to untreated cells, with only minor variations in normalized cell count and % confluency throughout the experimental period. In contrast, exposure to concentrations of 100 and 200 μg/mL resulted in inhibition of cell proliferation and a reduction in both cell count and normalized confluence relative to baseline, as indicated by stable or declining cell numbers over the 72 h monitoring period. These findings indicate a dose-dependent cytotoxic effect of HEE in HaCaT cells, with significant growth inhibition observed at concentrations exceeding 100 μg/mL.

Based on the dose–response curves, EC_10_ and EC_50_ values were estimated and are summarized in Table 1. The non-cytotoxic concentration of 20 μg/mL was therefore selected for use as the pre-treatment dose in subsequent experiments. Accordingly, the biological effects described in subsequent assays reflect responses at the specific concentration and do not establish a broader dose–response relationship across all functional endpoints.

### 2.3. Cytoprotective Effect of HEE Against Oxidative Agents in HaCaT Cell Line

**Figure 3:**
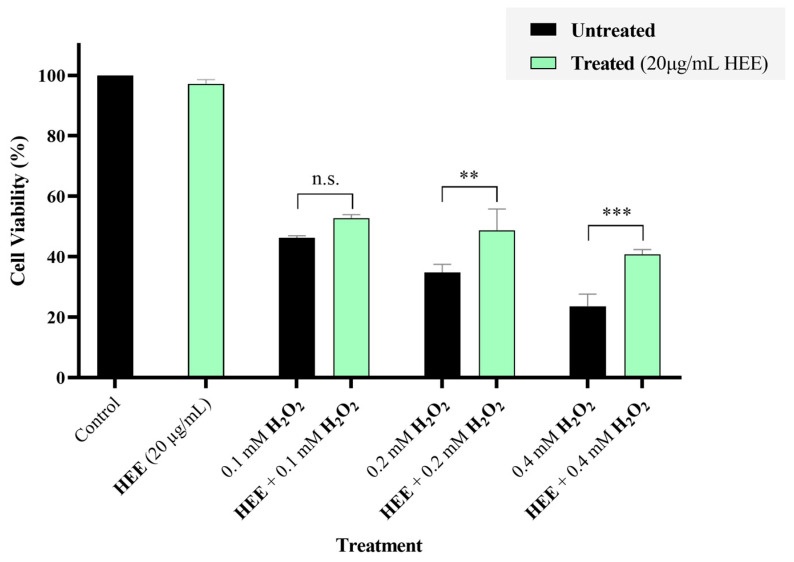
Cell-protective effects of HEE (20 μg/mL) against H_2_O_2_-induced oxidative stress in HaCaT cells. HaCaT cells were seeded in 96-well plates and pre-treated with HEE (20 μg/mL) for 24 h, followed by exposure to increasing concentrations of H_2_O_2_ for an additional 24 h. Cell viability was then evaluated using the SRB assay. Data are presented as mean ± SD (n = 3) from at least three independent experiments. Asterisks denote statistical significance of *p*-value (n.s., not significant (*p* ≥ 0.05), ** *p* < 0.01, *** *p* < 0.001) vs. corresponding control or H_2_O_2_-treated cells (HEE-untreated).

The cytoprotective potential of HEE in HaCaT cell line was evaluated against the oxidative stress inducer, H_2_O_2_. For this purpose, HaCaT cells were seeded in 96-well plates and pre-treated with HEE at the non-toxic concentration of 20 μg/mL for 24 h, followed by exposure to increasing concentrations of H_2_O_2_ for an additional 24 h. Cell viability was subsequently assessed using the SRB assay. As shown in Figure 3, pre-treatment with HEE resulted in higher cell viability compared with H_2_O_2_-treated cells across the tested concentrations, indicating a protective effect.

### 2.4. Effect of HEE on Intarcellular ROS Levels Assessed by H_2_DCFDA

**Figure 4:**
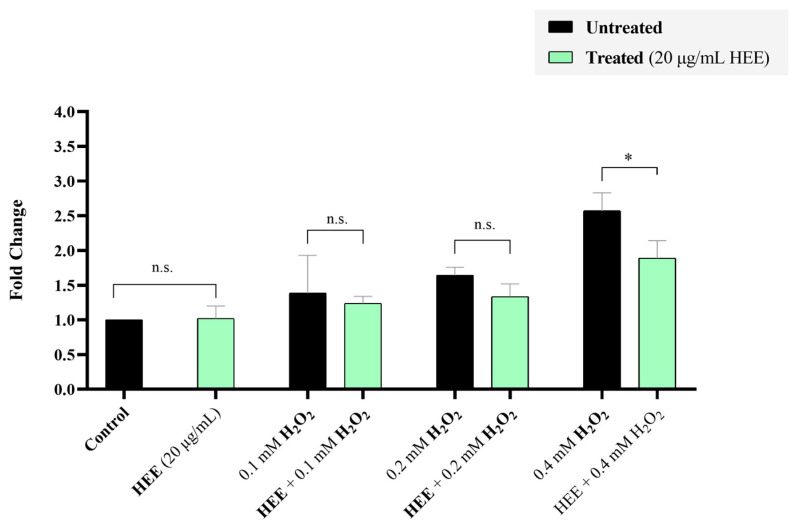
Effect of HEE on intracellular ROS levels in HaCaT cells exposed to H_2_O_2_-induced oxidative stress. Intracellular ROS levels were quantified using the H_2_DCFDA assay after 24 h treatment with H_2_O_2_ (0, 0.1, 0.2, 0.4 mM) in the presence or absence of HEE (20 μg/mL). Data are presented as mean ± SD (n = 3) from at least three independent experiments. Asterisks indicate statistical significance (* *p* < 0.05) vs. control or H_2_O_2_-treared cells (HEE-untreated); n.s., not significant.

HaCaT cells were treated for 24 h with H_2_O_2_ (0, 0.1, 0.2, 0.4 mM), HEE (20 μg/mL), or their combination to evaluate the antioxidant activity of HEE on intracellular ROS levels. As shown in Figure 4, exposure to H_2_O_2_ induced a concentration-dependent increase in intracellular ROS levels, with the strongest oxidative response observed at 0.4 mM. Co-treatment with HEE (20 μg/mL) significantly reduced intracellular ROS levels in H_2_O_2_-treated cells, indicating a cytoprotective effect. Across all oxidative conditions (0.1, 0.2, and 0.4 mM H_2_O_2_), HEE-treated cells displayed markedly lower fluorescence intensity compared with untreated cells.

### 2.5. Effect of HEE Wound Closure in HaCaT Cells

**Figure 5:**
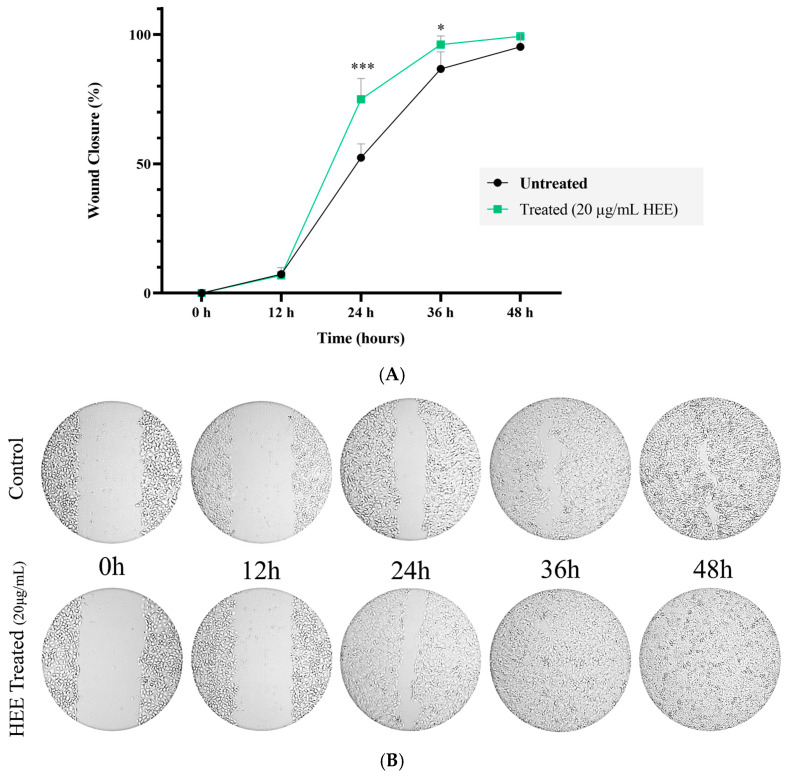
Effect of HEE on wound closure in HaCaT cells. (**A**) Quantitative analysis of wound closure in response to HEE treatment (20 μg/mL). Wound closure was monitored at 0, 12, 24, 36, and 48 h using a ZEISS Primovert Microscope (Zeiss, Göttingen, Germany) equipped with a digital camera (Axiocam ERc 5 s). The percentage of wound closure was calculated from captured images and analyzed for statistical significance. Data are presented as mean ± SD (n = 3). Asterisks denote statistical significance of *p*-value (* *p* < 0.05, *** *p* < 0.001) vs. control (untreated cells). (**B**) Representative images of the wound gap captured at 0, 12, 24, 36, and 48 h using a 4× objective.

The effect of HEE on wound closure in HaCaT cells was evaluated using a wound healing assay. As shown in Figure 5A,B, treatment with HEE (20 μg/mL) resulted in enhanced wound closure compared with untreated control cells, demonstrating a time-dependent effect. Notably, HEE-treated cells achieved complete closure of the wound gap by 36 h, whereas the control group displayed slower gap closure. These results indicate that HEE promotes wound closure in keratinocyte monolayers in vitro.

### 2.6. Effects of HEE on the Expression of Antioxidant Response Genes in HaCaT Cells

**Figure 6:**
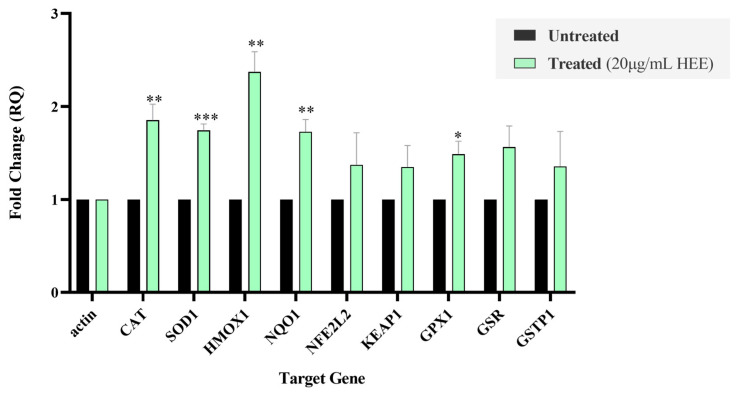
Relative gene expression of a panel of antioxidant-related genes in HaCaT cells following HEE treatment. HaCaT cells were treated with HEE (20 μg/mL) for 24 h, and mRNA expression levels were analyzed by RT-qPCR. Gene expression is presented as mean fold change relative to untreated control cells and was calculated using the comparative ∆∆ct method, with *β-actin* as the endogenous reference gene for normalization. Data are presented as mean ± SD (n = 3) from at least three independent experiments. Asterisks denote statistical significance of *p*-value (* *p* < 0.05, ** *p* < 0.01, *** *p* < 0.001) vs. control (untreated cells).

To determine whether HEE modulates the expression of oxidative stress-related genes, HaCaT cells were treated with HEE (20 μg/mL) for 24 h, followed by RNA isolation, cDNA synthesis, and quantitative real-time PCR analysis. Gene expression levels were normalized to the endogenous reference gene *β-actin*, and untreated cells were used as the calibrator control. As shown in Figure 6**,** HEE treatment resulted in an overall increase in the expression of the examined antioxidant-associated genes at the transcriptional level, with particularly significant increases observed for *CAT*, *SOD1*, *HMOX1*, *NQO1*, *GPX*, and *GSR*.

### 2.7. Transcriptomic Analysis via mRNA Sequencing of HEE-Treated HaCaT Cells

To investigate the global transcriptional response of HaCaT keratinocytes to HEE treatment, cells were treated with HEE (20 μg/mL) for 24 h, followed by mRNA sequencing (RNA-seq) analysis. Total RNA isolated from HEE-treated (20 μg/mL) and untreated HaCaT cells was subjected to high-throughput paired-end sequencing. Two biological replicates per condition were analyzed. After quality filtering and alignment to the human reference genome (GRCh38), differential gene expression analysis was conducted to identify genes significantly modulated by HEE exposure. Sequencing generated approximately 20 million paired-end reads per sample, of which the majority were successfully mapped to the reference genome (Untreated sample: 95.44% and Treated sample: 93.36%).

#### 2.7.1. Differentially Expressed Genes in Untreated and HEE-Treated HaCaT Cells

**Figure 7:**
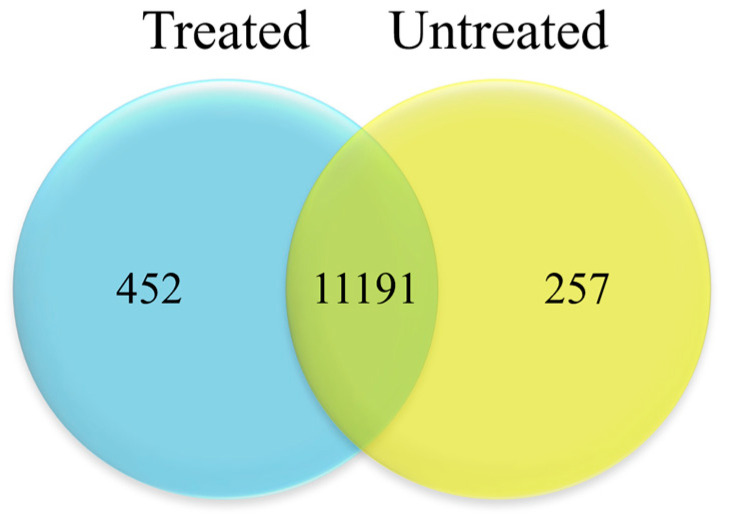
Venn diagram illustrating the co-expression of genes in untreated and HEE-treated HaCaT cells based on mRNA sequencing analysis. Overlapping regions indicate genes commonly expressed under both conditions, while non-overlapping regions represent genes uniquely expressed in untreated control cells or in response to HEE treatment.

To evaluate global transcriptional differences between HEE-treated and untreated HaCaT cells, comparative analysis of gene expression profiling was performed and visualized using a Venn diagram. As shown in Figure 7, the co-expression Venn diagram presents the number of genes uniquely expressed within each group/sample, as well as the genes shared between groups. The majority of genes were located in the overlapping region, suggesting a largely shared transcriptional landscape between treated and control cells. Nevertheless, a distinct subset of genes was uniquely expressed in HEE-treated cells, whereas a smaller number of genes were exclusively detected in untreated controls.

#### 2.7.2. Distribution of Differentially Expressed Genes Between HEE-Treated and Untreated HaCaT Cells

**Figure 8:**
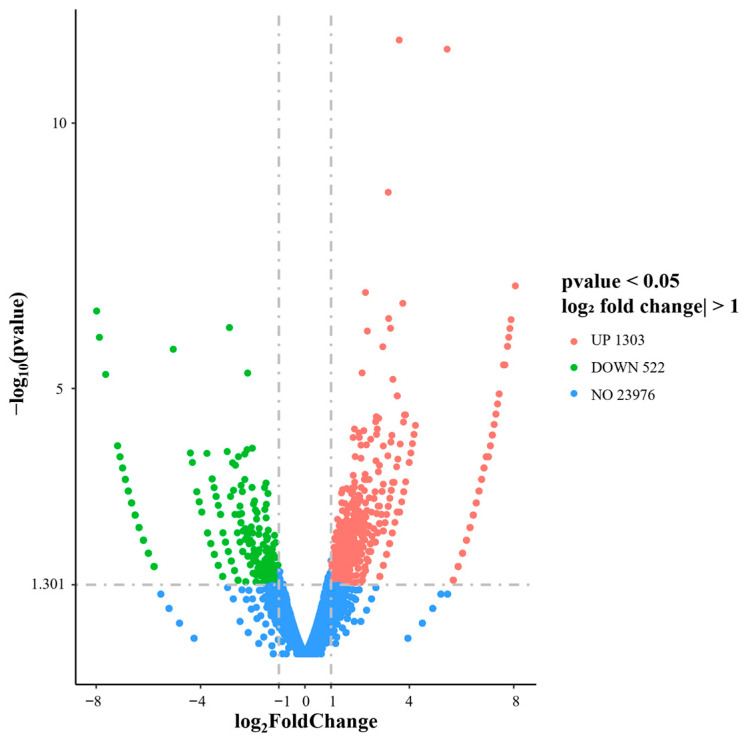
Volcano plot illustrating differentially expressed genes between untreated and HEE-treated HaCaT cells (20 μg/mL) as determined by RNA sequencing analysis. The *x*-axis represents log_2_ fold change, while the *y*-axis represents log_10_(*p*-value). Genes with |log_2_ fold change| ≥ 1 and *p*-value ≤ 0.05 were considered significantly differentially expressed. Upregulated genes are shown in red, downregulated genes in green, and non-significant genes in blue. Data are representative of two biological replicates.

Differential gene expression analysis between HEE-treated and untreated HaCaT cells revealed changes in gene expression associated with HEE treatment. As shown in Figure 8, a number of genes were significantly upregulated or downregulated following exposure to HEE (20 μg/mL), based on the threshold of |log_2_ fold change| ≥ 1 and *p*-value ≤ 0.05. Notably, HEE treatment resulted in a greater number of upregulated genes compared to downregulated genes, suggesting a bias toward increased gene expression programs. The majority of genes, however, did not exhibit statistically significant changes and clustered around log_2_ fold change values close to zero.

#### 2.7.3. Top 30 Up- and Down-Regulated Genes in HEE-Treated HaCaT Cells

**Figure 9:**
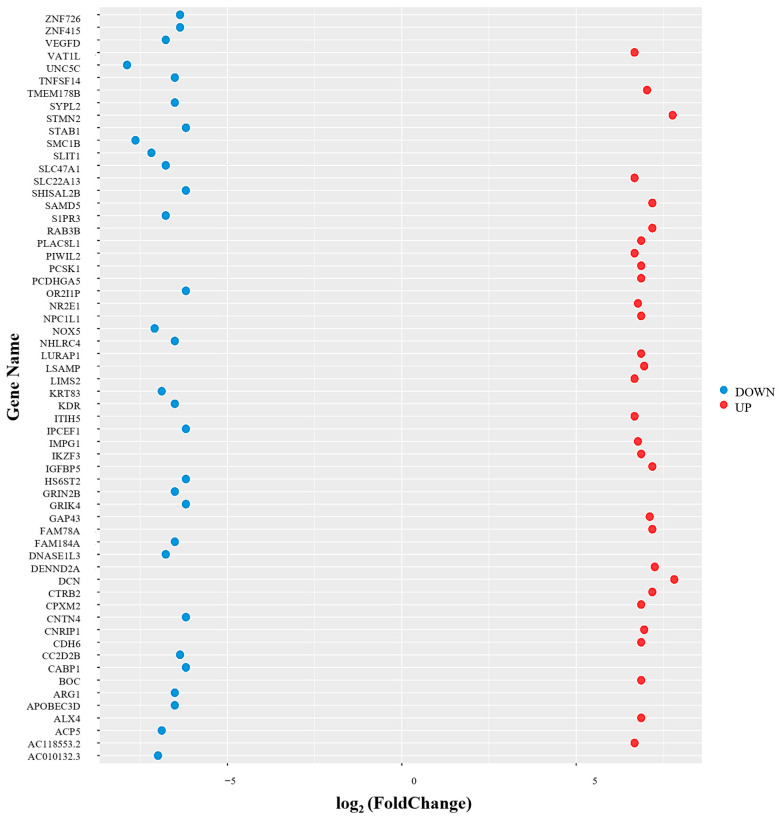
Dot plot depicting the top 30 upregulated and top 30 downregulated genes in HaCaT cells treated with HEE (20 μg/mL) compared to untreated cells, as identified by RNA sequencing analysis. The *x*-axis represents log_2_ fold change, while gene names are shown on the *y*-axis. Upregulated genes are indicated in red, and downregulated genes in blue. Data are representative of two biological replicates.

To further characterize the transcriptional changes associated with HEE treatment, the top 30 most upregulated and top 30 most downregulated genes were identified and are visualized in Figure 9 based on their log_2_ fold change values. The analysis showed a distribution of upregulated and downregulated gene sets, with several genes exhibiting notably increases or decreases in expression following HEE exposure. Upregulated genes displayed positive log_2_ fold change values, whereas downregulated genes showed negative log_2_ fold changes, reflecting changes in gene expression associated with HEE treatment.

#### 2.7.4. Heatmap of Selected Differentially Expressed Genes in HEE-Treated HaCaT Cells

**Figure 10:**
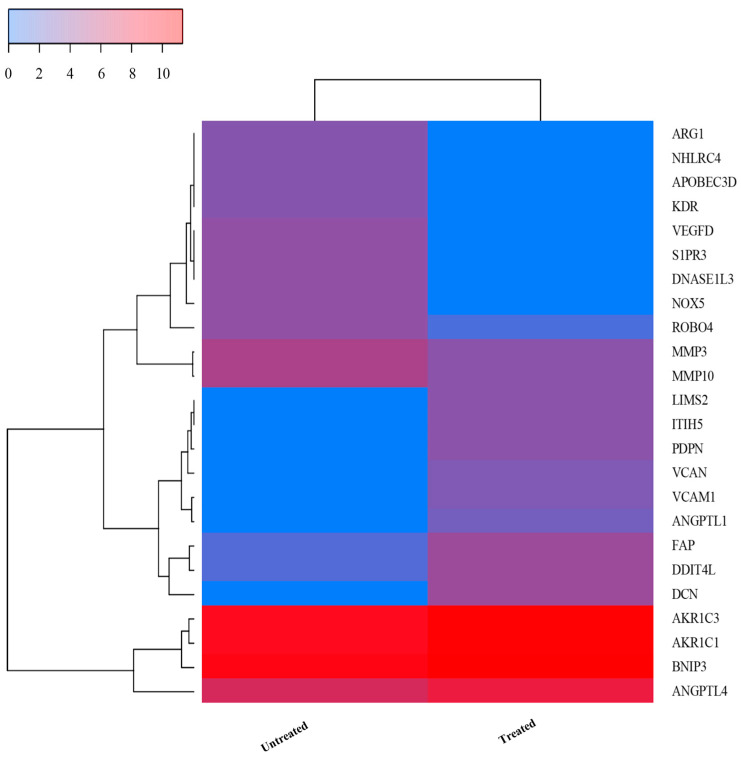
Heatmap of selected differentially expressed genes associated with ECM remodeling, oxidative stress response, DNA damage, and cell survival pathways in HaCaT cells treated with HEE (20 μg/mL) compared to untreated cells, as determined by RNA sequencing analysis. Gene expression values were log_2_-transformed [log_2_(value + 1)] and subjected to hierarchical clustering. Color intensity indicates relative expression levels, with blue indicating lower and red indicating higher expression. Data are representative of two biological replicates.

To further explore gene expression patterns related to oxidative stress response, extracellular matrix remodeling, and DNA damage-related pathways, a heatmap analysis (Figure 10) was performed on a selected subset of differentially expressed genes exhibiting high fold change. Hierarchical clustering suggested a separation between treated and untreated samples, indicating differences in gene expression patterns following HEE exposure.

#### 2.7.5. KEGG Pathway Enrichment

**Figure 11:**
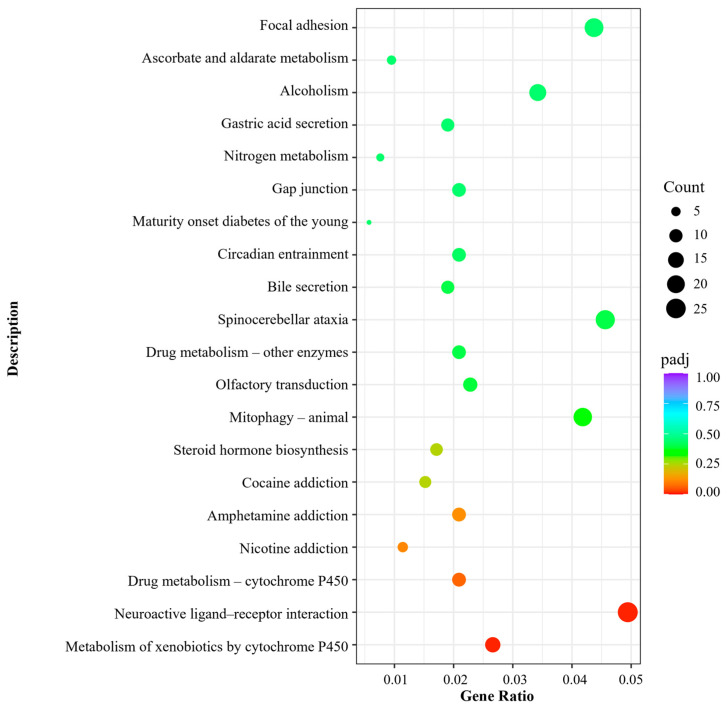
KEGG pathway enrichment analysis of differentially expressed genes in HaCaT cells following treatment with HEE (20 μg/mL). Dot plot illustrating the top 20 enriched pathways based on gene ratio (abscissa), gene count (dot size), and adjusted *p*-value (color scale). Enrichment analysis was performed using a hypergeometric statistical model with Benjamini–Hochberg false discovery rate (FDR) correction. Data are representative of two biological replicates.

KEGG pathway enrichment analysis was performed to identify biological pathways associated with the differentially expressed genes in HEE-treated HaCaT cells. As shown in Figure 11, several pathways were enriched following treatment, with a subset reaching statistical significance, including xenobiotic and drug metabolism, steroid hormone biosynthesis, mitophagy, extracellular matrix organization, gap junction signaling, and metabolic adaptation pathways. Notably, multiple enriched categories were related to cytochrome P450–mediated metabolism, suggesting a potential association with cellular detoxification-related processes.

#### 2.7.6. Reactome Pathway Enrichment

**Figure 12:**
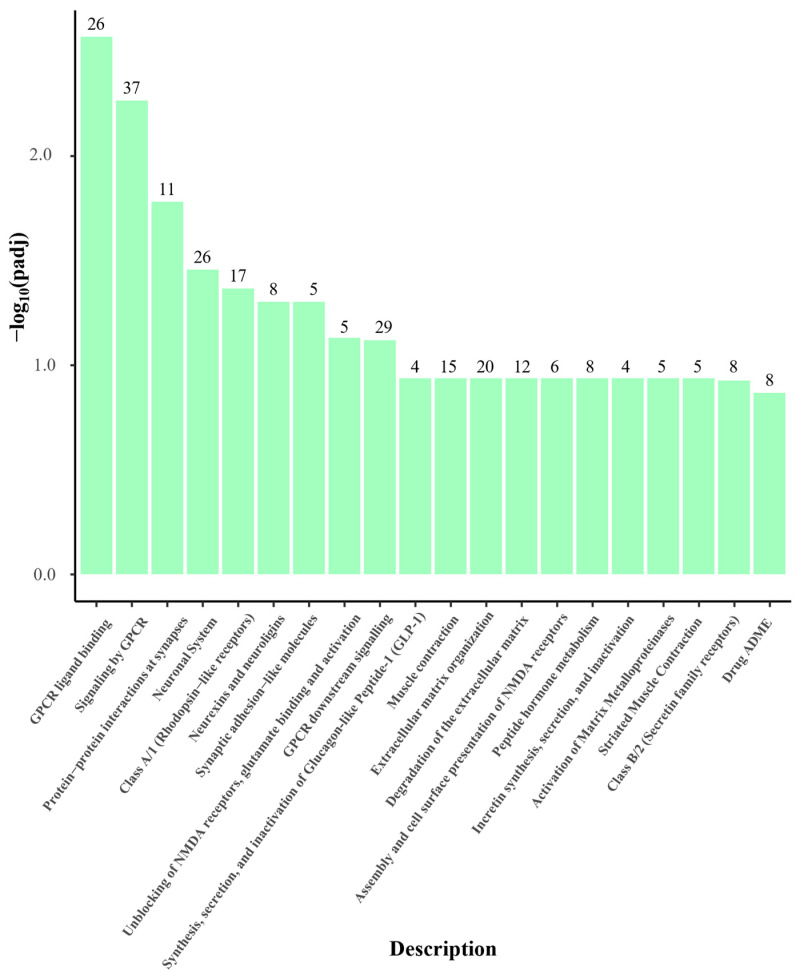
Reactome pathway enrichment analysis of differentially expressed genes in HaCaT cells treated with HEE (20 μg/mL) compared to untreated cells. The bar plot displays the top 20 enriched biological pathways, ranked according to −log_10_(adjusted *p*-value). Numbers above each bar indicate the number of genes associated with each pathway. Pathway enrichment was performed using a hypergeometric over-representation test, and *p*-values were corrected for multiple testing using the Benjamini–Hochberg false FDR method. Data are representative of two biological replicates.

Reactome pathway enrichment analysis was conducted to further explore the biological pathways associated with the transcriptional changes induced by HEE treatment in HaCaT cells. As visualized in Figure 12, several pathways related to cell communication and signaling regulation were enriched following treatment, with a subset reaching statistical significance, including G protein-coupled receptor (GPCR) ligand binding, signaling by GPCR, protein–protein interactions at synapses, and neuronal system-related modules. Additional enriched categories included extracellular matrix organization, peptide hormone metabolism, and activation of matrix metalloproteinases, suggesting potential associations with tissue remodeling- and wound-healing-related responses.

## 3. Discussion

The findings of this study provide evidence that the ethanolic extract of *Haberlea rhodopensis* exerts antioxidant and cytoprotective effects and is associated with pro-regenerative responses in human keratinocytes subjected to oxidative stress. This aligns with prior work where HEE reduces ROS, activates antioxidant defenses, and preserves cell integrity under oxidative stress or radiation-induced stress in both cell-free and cellular models [18,19]. Similar protective responses have been reported for other plant-derived compounds in keratinocytes [20,21,22].

It is important to note that the biological effects observed in our study arise from the use of a crude total extract and therefore cannot be attributed to a single compound, but rather to the synergistic action of multiple phytochemicals. The combination of phenolic acids, flavonoids, and terpenoids may enhance bioavailability, stability, and pathway activation, resulting in a more pronounced biological response compared to individual constituents alone [6]. Such combinatorial effects are well documented for polyphenolic compounds, which act not only as direct antioxidants but also as modulators of multiple cellular signaling pathways, particularly in keratinocyte and other skin models [9,18,20]. The ability of HEE to protect plasmid DNA from oxidative damage aligns with its high phenolic and flavonoid content [5] and corroborates previous reports on the plant’s radical-scavenging properties. This DNA-protective activity extends these observations by demonstrating direct protection in a cell-free system [10,19,21], reflecting the antioxidant potential of HEE in limiting oxidative macromolecular damage under cell free conditions [12,23]. Phenolic acids and flavonoid derivatives previously identified in HEE, including caffeic, chlorogenic and rosmarinic acids, as well as quercetin glycosides [5], may contribute to these effects through their well-established ROS-neutralizing properties and redox-modulating capacity [5,9].

At the cellular level, the concentration-dependent effects of *Haberlea* extracts on HaCaT keratinocyte viability and proliferation reflect the dual nature of these phytocompounds, whereby optimal doses confer cytoprotection, while higher concentrations exert significant antiproliferative effects. These effects may be mediated by changes at the cell periphery through membrane permeabilization, actin cytoskeleton reorganization, and the disruption of tight junctions in actively dividing cells [5,22,24]. This biphasic response parallels findings in cancer cell lines, where the ethanolic extract displays dose-dependent cytotoxic and antimigratory effects [5].

The selection of a non-toxic pre-treatment concentration of HEE (20 μg/mL) enabled the demonstration of enhanced keratinocyte survival under oxidative challenge, as shown by SRB viability assays following exposure to increasing concentrations of H_2_O_2_. All functional assays were conducted at a single non-cytotoxic concentration (20 μg/mL); therefore, the observed effects are specific to this dose and do not establish a broader dose–response relationship across all endpoints. Compared with cells exposed to H_2_O_2_ alone, pre-incubation with HEE preserved cell viability across stress conditions, suggesting a protective mechanism beyond simple cytotoxic attenuation. This dual mode of action likely involves interaction with extracellular ROS and/or modulation of cellular redox responses [20,25]. On one hand, HEE could contribute to potential interactions with reactive species, thereby limiting the initial oxidant burden. On the other hand, its protective action may involve pre-conditioning and reinforcement of endogenous antioxidant and stress-response pathways, contributing to the maintenance of redox balance during subsequent oxidant exposure—a pattern that has been similarly reported for other plant-derived bioactive extracts in keratinocytes [18,20,23]. The complementary DCFDA assay further supports this interpretation. Lower intracellular ROS accumulation in HEE-pretreated cells indicates either reduced ROS generation or improved cellular handling of ROS, in agreement with the preservation of cell viability. Taken together, these results indicate that HEE does not simply prevent H_2_O_2_-induced cytotoxic damage, but may instead contribute to the preservation of cellular redox homeostasis through combined antioxidant and cell-protective actions [20,25]. In this context, phenolic acids may contribute to the direct neutralization of extracellular reactive species, while flavonoid derivatives can support intracellular antioxidant defenses through modulation of redox-sensitive signaling pathways, thereby providing a mechanistic basis for the reduced ROS accumulation observed in HEE-treated cells [9,19]. Similar protective effects of related secondary metabolites have also been reported in keratinocyte and fibroblast systems exposed to oxidative stress [20,23,25].

Beyond cytoprotection, the pro-regenerative potential of HEE, evidenced by enhanced closure in keratinocyte monolayers, underscores the extract’s capacity to support skin repair processes [26,27]. It should be noted that this assay does not distinguish between cell migration and proliferation, and therefore the observed wound closure likely reflects a combination of these processes. The interplay between oxidative stress modulation and wound healing is well-recognized, with excessive ROS known to impair tissue regeneration [28]. By establishing an antioxidant environment, HEE preserves keratinocyte functionality and promotes reparative mechanisms, reflecting the intricate balance required for effective migration and wound healing [18,28,29]. Phenolic and flavonoid constituents, as well as specific components such as the phenylethanoid glycoside myconoside, may support these responses by modulating oxidative stress and preserving cellular function under challenging conditions [30,31]. In line with this, myconoside-rich extracts of *H. rhodopensis* have been reported to enhance antioxidant defenses and stimulate extracellular matrix protein synthesis, including collagen and elastin, further supporting their relevance in skin repair [32]. These findings echo observations with other botanical extracts that enhance keratinocyte motility and proliferation, reinforcing the therapeutic potential of plant-derived antioxidants in dermatological applications [18,25,33].

The observed cellular activities resonate with the survival strategies of *H. rhodopensis* as a resurrection plant, which employs robust antioxidant defenses and precise transcriptional regulation to withstand dehydration–rehydration cycles [19,30,32]. Therefore, interventions that normalize oxidative load can favor wound closure kinetics [28]. Our findings suggest that these evolutionarily conserved protective mechanisms can be functionally translated to human skin cells, highlighting *H. rhodopensis* as a valuable source of bioactive compounds for human skin health and protection, particularly in the context of environmental stress and aging [12,32,34].

At the molecular level, HEE treatment upregulated key antioxidant genes such as *CAT*, *SOD1*, *GPX1*, *GSR*, *HMOX1*, and *NQO1*, indicating coordinated modulation of redox homeostasis [19,23,35]. The concurrent modulation of NFE2L2 (NRF2) and Keap1 is consistent with modulation of the canonical NRF2 regulatory axis, a well-established pathway governing antioxidant gene expression; however, direct functional validation of NRF2 activation (e.g., nuclear translocation of protein-level induction of downstream targets) was not performed in the present study. This observation aligns with established mechanisms whereby plant polyphenols have been reported to modulate NRF2/Keap1 signaling to enhance endogenous antioxidant capacity and restore redox balance [36,37,38]. A mechanistic precedent for NRF2-centered protection in HaCaT cells has been previously reported for *H. rhodopensis*-derived constituents (including myconoside and calceolarioside E) in UV-stressed keratinocytes, where NRF2 activation was associated with downstream induction of *SOD* and *CAT* and improved oxidative recovery [19,31]. Similar NRF2/HO-1-linked cellular responses have also been documented for other plant-derived extracts in keratinocytes [18,39,40], supporting the plausibility of HEE promoting resilience via transcriptional remodeling of redox-defense pathways rather than through a single stress-response mechanism.

The transcriptomic profile obtained by mRNA sequencing of HaCaT cells exposed to HEE indicates a selective, condition-dependent modulation of gene expression rather than global transcriptional disruption. Most core genes remained expressed in both treated and untreated cells, indicating preserved cellular functions and arguing against nonspecific transcriptional toxicity. Instead, the presence of treatment-specific transcripts and the asymmetric distribution of differentially expressed genes suggest a shift in gene expression patterns consistent with adaptive or compensatory responses described in epithelial and wound-healing contexts exposed to plant-derived polyphenols or multicomponent natural preparations [36,37,38]. Similar patterns of selective transcriptome reshaping have been reported in murine and human wound-healing RNA-seq and multi-omic studies, where differential regulation occurs predominantly in pathways related to keratinization, ECM organization, immune signaling, and inflammation, while basal cellular programs remain largely intact [41,42,43]. Notably, upregulation of genes such as *AKR1C1*, *AKR1C3*, *BNIP3*, *ANGPTL4*, *PDPN*, and *VCAM1*, together with the downregulation of transcripts including *ARG1*, *VEGFD*, and *NOX5*, may reflect adjustments in metabolism, ECM interactions, and cell-microenvironment communication networks that become relevant under oxidative challenge and wound-healing-associated remodeling. Comparable transcriptional signatures involving metabolic rewiring, ECM and adhesion dynamics, and inflammatory tone have been reported in studies distinguishing acute from chronic wounds, regenerative versus scarring trajectories, and keratinocyte state transitions during repair [42,44,45]. At the pathway level, KEGG and Reactome enrichment analysis highlighted pathways related to ECM organization, adhesion, and signaling, consistent with changes in gene expression patterns, aligning with pathway-level remodeling observed in natural-product-treated keratinocytes and in vitro wound-healing models [33,46,47].

Overall, HEE appears to induce a recovery-supportive or pro-remodeling transcriptional state rather than broad cytotoxic stress. However, the observed transcriptomic trends should be interpreted as indicators of potential adaptive or compensatory responses, not as definitive proof of functional activation of wound-healing or stress-resilience mechanisms. A limitation of this study is that the transcriptomic analysis was based on two biological replicates per condition; therefore, these findings should be considered exploratory and hypothesis-generating. Further validation at the protein and functional levels, including oxidative stress and redox-balance markers, ECM remodeling assays, keratinocyte migration and scratch-wound-closure models, and integration with multi-omic readouts, will be required to determine whether the transcriptomic modulation observed here translates into measurable regenerative phenotypic outcomes [42,43,44,48]. Additionally, direct measurements of antioxidant activity and kinetic characterization (e.g., Km, Vmax), as well as protein-level validation of key pathways (e.g., NRF2 signaling and downstream targets) and time-resolved assessment of ROS dynamics, would provide deeper mechanistic insight. Overall, the mechanistic interpretations proposed here should be considered indicative and provide a foundation for further investigation.

Taken together, our findings support a model in which HEE provides multi-layered protection: (i) antioxidant activity as evidenced by protection against oxidative DNA damage in a cell-free system, (ii) reduction in stress-induced intracellular ROS accumulation, and (iii) transcriptional modulation of antioxidant and detoxification genes, consistent with potential engagement of endogenous defense pathways. This integrated profile extends previous observations of *H. rhodopensis* extracts, which can tune stress responses and modulate gene expression following oxidative and genotoxic insults, while extending these findings to a skin-relevant keratinocyte model and supporting responses associated with wound closure [19,35]. Future studies should aim to validate pathway engagement at the protein level (e.g., NRF2 nuclear translocation, HO-1/NQO1 protein induction), evaluate longer exposure windows and repeated-stress paradigms, further characterize the contribution of specific bioactive constituents of HEE to the observed effects, and explore performance in advanced skin models to further support translational applications [34,49].

## 4. Materials and Methods

### 4.1. Chemicals and Reagents

Dulbecco’s modified Eagle’s medium (DMEM) high glucose, Trypsin-EDTA, penicillin–streptomycin and phosphate-buffered saline (PBS) were obtained from Biosera (Cholet, France). Fetal bovine serum (FBS) was purchased from Gibco (Waltham, MA, USA). Hydrogen peroxide, sulforhodamine B andiron (II) sulfate heptahydrate were purchased from Sigma-Aldrich (St. Louis, MO, USA). Trichloroacetic acid (TCA) and Tris ACS ultra-pure were obtained from AppliChem (ITW Reagents, Milano, Italy). Glacial acetic acid and isopropanol were purchased from Scharlau (Barcelona, Spain). Agarose (multiple purpose) was obtained from MP Biomedicals (Illkirch-Graffenstaden, France), and low-EEO agarose was purchased from Thermo Scientific Chemicals (Waltham, MA, USA). Ethidium bromide was obtained from Biotium, Inc. (Fremont, CA, USA). 2′,7′-Dichlorodihydrofluorescein diacetate (H_2_DCFDA/DCF-DA) and the SuperScript First-Strand Synthesis System for RT-qPCR were obtained from Thermo Fisher Scientific (Waltham, MA, USA). KAPA SYBR Fast Master Mix was purchased from Kapa Biosystems (Hoffmann-La Roche, Basel, Switzerland). Primers were synthesized by Eurofins (Val Fleuri, Luxembourg). NucleoZOL was obtained from Macherey-Nagel (Düren, Germany), and the RNeasy Mini Kit was purchased from Qiagen (Hilden, Germany). pUC19 plasmid DNA was obtained from Addgene (Watertown, MA, USA).

### 4.2. Plant Material and Preparation of Ethanol Extract

*Haberlea rhodopensis* plants were routinely propagated in vitro and adapted to pots under controlled conditions, as previously described by Djilianov et al. [50]. Fully developed leaves from well-hydrated plants were collected, detached, and air-dried prior to extraction. For the HEE, 50 mg of dried leaves were finely ground and incubated with 0.5 mL of 70% ethanol for 48 h at room temperature. After centrifugation at 10,000× *g* for 10 min, the pellet was re-extracted with ethanol for an additional 24 h under the same conditions. The two supernatants were pooled and evaporated at 40 °C using a SpeedVac concentrator (Labconco Corporation, Kansas City, MO, USA). The obtained dry extract was stored at −20 °C until further use. For cell treatments, HEE was freshly dissolved in 20% dimethyl sulfoxide (DMSO), prepared in culture medium, and incubated with cells at 37 °C to ensure complete solubilization and stability before application.

### 4.3. Plasmid DNA Protection Assay

The plasmid DNA protection assay was used to assess the ability of HEE to prevent oxidative damage induced by a Fenton reaction system (FeSO_4_/H_2_O_2_), which generates highly reactive hydroxyl radicals (•OH) that convert supercoiled plasmid DNA into its linear and relaxed forms. For this assay, the pUC19 plasmid was used at a final concentration of 100 ng/μL. The Fenton reagent was freshly prepared to yield final concentrations of 10 μM FeSO_4_ and 1 mM H_2_O_2_ in the reaction mixture. HEE was tested at graded concentrations (0.002, 0.02, 0.2, 2, 4, and 5 mg/mL), and the final reaction volume was adjusted to 20 μL with ultrapure water. The order of addition was as follows: ddH_2_O → pUC19 DNA → HEE → FeSO_4_ → H_2_O_2_. After gentle mixing, samples were incubated at 37 °C for 30 min and then mixed with 2 μL of 1× loading buffer (0.25% bromophenol blue in 50% glycerol). Reaction mixtures were analyzed by electrophoresis on 0.8% agarose gels prepared in 1× TAE buffer containing ethidium bromide (0.5 μg/mL). Electrophoresis was performed at 90 V for 1 h, and DNA bands were visualized under UV illumination using a gel documentation system (Bio-Rad, Hercules, CA, USA). Plasmid conformations were distinguished as supercoiled, linear, or relaxed circular according to their migration pattern. All experiments were performed in three (3) independent replicates.

### 4.4. Cell Culture

The human cell line HaCaT (immortalized human epidermal keratinocytes) was obtained from the American Type Culture Collection (ATCC) (Rockville, MD, USA). Cells were maintained under sterile conditions at 37 °C in a humidified atmosphere of 5% CO_2_. They were routinely passaged with trypsin and cultured in DMEM medium supplemented with 10% FBS and 1% penicillin–streptomycin (100 U/mL penicillin and 100 µg/mL streptomycin).

### 4.5. Cell Treatments

HaCaT cells were seeded at appropriate densities in culture plates, depending on the assay type and selected time point, in DMEM medium supplemented with 10% FBS and allowed to adhere overnight under standard cell culture conditions (37 °C, 5% CO_2_). The following day, cells were washed with PBS and treated with either serial dilutions of HEE (final DMSO concentration ≤ 0.1% *v*/*v*) or pre-treated with the non-cytotoxic EC_10_ concentration of 20 μg/mL. Control wells were treated with medium only (containing the corresponding DMSO concentration).

### 4.6. Cell Proliferation Assay

Cell proliferation and viability were assessed using the colorimetric SRB assay, a method that enables reliable quantification of total cellular protein content as an indirect measure of cell density. HaCaT cells were seeded in 96-well plates at 5.0 × 10^3^ cells/well, and allowed to adhere overnight (37 °C, 5% CO_2_). The following day, cells were washed with phosphate-buffered saline (PBS) and either treated with serial dilutions of HEE for 48 or 72 h to assess cytotoxicity, or pre-treated for 24 h with a non-toxic concentration of HEE (EC_10_ = 20 μg/mL), followed by an additional 24 h exposure to increasing concentrations of H_2_O_2_ to evaluate cytoprotective effects under oxidative stress. Wells containing only culture medium (and solvent when applicable) were processed in parallel and used as blanks.

After incubation, cells were fixed with 50% TCA, stained with 0.4% *w*/*v* SRB, and resolved with 10 mM Tris base. Absorbance was measured at 570 nm using a microplate reader (Enspire, Perkin Elmer, Waltham, MA, USA). Each treatment was tested in at least three (3) technical replicates, and all experiments were independently repeated at least three (3) times. Cell survival (%) was calculated using the following formula:

(1) $$\%\;\mathrm{Viability}=\;\frac{{OD}_{540}\;\mathrm{Sample}-\;{OD}_{540}\;\mathrm{Blank}}{{OD}_{540}\;\mathrm{Control}\;-\;{OD}_{540}\;\mathrm{Blank}}\;\times \;100$$

where

*OD_Control_ = Optical Density of Untreated Cells*

*OD_Blank_ = Optical Density of Blank Wells*

### 4.7. HoloMonitor^®^ M4 Cell Proliferation Assay

HaCaT cells were seeded in clear-bottom 96-well plates (SPL Life Sciences, Pocheon, South Korea) fitted with HoloMonitor lids at 4–5 × 10^3^ cells/well (corresponding to approximately 10% initial frame coverage) and allowed to adhere overnight under standard culture conditions. The following day, cells were washed with PBS and treated with serial dilutions of HEE. The plate was then placed in the HoloMonitor^®^ M4 system (Phase Holographic Imaging AB, Lund, Sweden), housed within a cell culture incubator. Cell imaging was performed at 2 h intervals over a total period of 72 h. Cell counts (normalized to area) were quantified from ≥3 distinct frame positions per well using HStudio™ software version 4.0.2 (Phase Holographic Imaging AB, Lund, Sweden).

### 4.8. 2′,7′-Dichlorodihydrofluorescein Diacetate (H_2_DCFDA/DCF-DA) Assay

The 2′,7′-dichlorodihydrofluorescein diacetate (H_2_DCFDA/DCF-DA) assay was used to detect intracellular ROS based on the oxidation of the non-fluorescent probe H_2_DCFDA into the highly fluorescent compound dichlorofluorescein (DCF). HaCaT cells were seeded in 96-well black-walled, clear-bottom plates at a density of 5 × 10^3^ cells/well and allowed to adhere overnight under standard culture conditions. The following day, cells were treated with HEE (20 μg/mL), H_2_O_2_ (0, 0.1, 0.2, and 0.4 μM), or combinations of HEE and H_2_O_2_. Following treatment, cells were washed with PBS and incubated with 100 μL H_2_DCFDA (20 μM), prepared in serum-free, phenol-red-free medium for 30–45 min at 37 °C in the dark. After incubation, cells were washed twice with PBS and replaced with fresh serum-free medium. Fluorescence was immediately measured using a microplate reader (Enspire, Perkin Elmer) at excitation/emission wavelengths of 485/535 nm. All assays were carried out under reduced light conditions to prevent probe degradation, and each experiment was performed in at least three (3) independent replicates. Fluorescence values were normalized and expressed as fold-change relative to untreated control cells.

### 4.9. Wound Healing Assay

For the wound healing assay, HaCaT cells were seeded in 6-well plates at an initial density of 2.5–3.0 × 10^5^ cells/well. Once full confluence was reached, the culture medium was removed, and the monolayers were gently washed with PBS. A straight linear scratch was generated in the center of each well using a sterile 200 μL pipette tip. Detached cells and debris were removed by an additional PBS wash. Cells were then treated with HEE (20 μg/mL), while untreated cells served as controls. Images of the wound area were captured at 0, 12, 24, 36, and 48 h using a ZEISS Primovert light microscope (Zeiss, Göttingen, Germany) equipped with a digital camera (Axiocam ERc 5 s), using a 4× objective, keeping the same field of view for each time point. Quantitative analysis of wound closure was performed by measuring the wound-gap width at each time point using the acquired micrographs. Percentage wound closure was calculated using ImageJ version 1.54s (NIH, Bethesda, MD, USA) with the Wound healing size macro tool as designed and described by Suarez-Arnedo et al. [51]. For each time point, the wound area was measured, and the % percentage of wound closure was calculated as the change in gap width compared to the corresponding initial wound width at 0 h. All conditions were assessed in at least three (3) independent experiments.

### 4.10. Real-Time PCR

Gene expression analysis was performed by quantitative real-time PCR (RT-qPCR) to evaluate the transcriptional response of antioxidant pathways in HaCaT cells following treatment with HEE. HaCaT cells were seeded in 60-mm dishes (1–1.5 × 10^6^ cells/dish) and treated with HEE (20 μg/mL) for 24 h. Total RNA was isolated using NucleoZOL reagent according to the manufacturer’s instructions, and RNA concentration and purity were assessed spectrophotometrically using a NanoDrop™ 1000 Spectrophotometer (Thermo Scientific). One microgram of total RNA was reverse-transcribed into cDNA using the SuperScript™ IV First-Strand Synthesis System with random hexamer primers. RT-qPCR reactions were performed using the SYBR^®^ FAST qPCR Kit on an ABI StepOne™ Real-Time PCR System (Applied Biosystems™, Thermo Fisher Scientific, Waltham, MA, USA). Reactions were set up in 96-well optical PCR plates (Applied Biosystems™, Thermo Fisher Scientific) and sealed with optical adhesive film (Applied Biosystems™, Thermo Fisher Scientific). The expression levels of *CAT* (Catalase), *SOD1* (superoxide dismutase-1), *HMOX1* (heme oxygenase 1), *NQO1* (NAD(P)H quinone dehydrogenase 1), *NFE2L2* (NRF2 nuclear factor erythroid 2-related factor 2), *Keap1* (Kelch-like ECH-associated protein 1), *GPX1* (glutathione peroxidase 1), *GSR* (glutathione reductase) and *GSTP1* (glutathione S-transferase pi-1) were analyzed, with *β-actin* used as the housekeeping gene for normalization. Relative gene expression was calculated using the 2^−ΔΔCt^ method and expressed as fold change relative to untreated control cells. All reactions were performed in triplicate, and no-template controls were included to confirm assay specificity. Primer sequences used for RT-qPCR are listed in Table 2.

### 4.11. Transcriptomic Analysis by mRNA Sequencing

HaCaT cells were seeded in 60-mm dishes at a density of approximately 5 × 10^5^ cells per dish and treated with HEE (20 μg/mL) for 24 h. Following treatment, cells were harvested by trypsinization, and total RNA was isolated using the RNeasy Mini Kit according to the manufacturer’s instructions. RNA quantity and integrity were assessed before library preparation. Poly(A)+ mRNA was enriched from total RNA using oligo-dT magnetic beads, followed by RNA fragmentation, first-strand cDNA synthesis using random hexamer primers, and second-strand cDNA synthesis. Libraries were constructed through end repair, A-tailing, adaptor ligation, size selection, PCR amplification, and purification, and were quality-controlled using Qubit fluorometer (Thermo Fisher Scientific, Waltham, MA, USA), real-time PCR (Thermo Fisher Scientific, Waltham, MA, USA) and Bioanalyzer analysis (Agilent Technologies, Santa Clara, CA, USA). Following library validation, samples were pooled and sequenced on an Illumina NovaSeq 6000 platform (Illumina, San Diego, CA, USA) using paired-end 150-bp reads, generating approximately 20 million reads per sample (~6 Gb per sample). Raw reads were processed with FastQC (v0.74) [52], read trimming using Trimmomatic (v0.39) [53] to remove adapter sequences, low-quality reads, and reads containing poly-N stretches, and quality metrics, including Q20, Q30, and GC content, were calculated. Filtered reads were aligned to the human reference genome (GRCh38) using HISAT2 (v2.2.1) [54] with splice-aware alignment. Transcript assembly was performed using StringTie (v2.1.5) [55] and gene-level read counts were obtained with featureCounts (v2.0.3) [56]. For expression level estimation and visualization purposes, read counts were normalized using fragments per kilobase of transcript per million mapped reads (FPKM). Differential gene expression analysis between HEE-treated and control samples was conducted using DESeq2 (v1.1.0) [57] with Benjamini–Hochberg false discovery rate (FDR) correction (adjusted *p* < 0.05, |log_2_ fold change| > 1). Functional enrichment analyses of differentially expressed genes were performed using Kyoto Encyclopedia of Genes and Genomes (KEGG) [58] and Reactome databases [59]. In addition, Gene Set Enrichment Analysis (GSEA) was performed with EGSEA (v1.20.0) [60] and applied to identify significantly enriched biological pathways and regulatory networks, and volcano plot, heatmap2, and scatterplot with ggplot2 [61] for visualization.

### 4.12. Statistical Analysis

Statistical analyses were performed using GraphPad Prism version 8.0 (GraphPad Software, San Diego, CA, USA). Data were first evaluated for normality using the Shapiro–Wilk test. Depending on data distribution and experimental design, appropriate parametric or non-parametric statistical tests were applied, including *t*-tests and analysis of variance (ANOVA) models, together with their corresponding multiple-comparison correction procedures (Holm–Sidak, Tukey, or Dunnett tests).

Statistical analysis of mRNA sequencing data was performed using the web-based Galaxy platform (usegalaxy.org) [62], which was employed for quality control of raw reads using FastQC (v0.74), read trimming with Trimmomatic (v0.39), alignment to the human reference genome (GRCh38) using HISAT2, read quantification with featureCounts, and differential gene expression and statistical analysis using DESeq2 with the appropriate statistical criteria.

All quantitative results are presented as mean ± standard deviation (SD), and statistical significance was defined as *p* < 0.05. Where applicable, results were expressed using the appropriate units or transformations (e.g., log_2_ fold change, −log_10_ *p*-value, log_2_(value + 1)), and *p*-values were adjusted for multiple testing using the Benjamini–Hochberg false discovery rate (FDR) correction.

## 5. Conclusions

This study demonstrates that the ethanolic extract of *Haberlea rhodopensis* exerts antioxidant and cytoprotective effects and is associated with wound-closure-associated responses in HaCaT keratinocytes under oxidative stress conditions. HEE reduced intracellular ROS levels and preserved cell viability following H_2_O_2_ exposure, indicating a protective effect. Moreover, wound healing assays revealed enhanced wound closure in keratinocyte monolayers in response to HEE treatment. At the molecular level, transcriptomic analysis indicated modulation of key antioxidant-response genes, consistent with potential engagement of endogenous redox-defense pathways. Complementary mRNA-sequencing further revealed a selective, stress-adaptive modulation of transcriptional programs without disruption of basal cellular functions, involving pathways related to extracellular matrix interactions, metabolic adaptation, and tissue repair-associated signaling. Collectively, these results suggest that HEE enhances cellular resilience and is associated with processes linked to oxidative protection and wound closure at non-toxic concentrations. This profile highlights the potential relevance of *H. rhodopensis* as a source of bioactive compounds for future dermatological, pharmaceutical, and cosmeceutical applications, while underscoring the need for further mechanistic studies and in vivo validation.

## Figures and Tables

**Table 1:** EC_10_ and EC_50_ values determined from cytotoxicity dose–response analyses in HaCaT cells. Data represent the concentrations at which the tested compound induces 10% and 50% reduction in cell viability. Values are presented as mean ± SEM (n = 3) of at least three experiments and were estimated using a nonlinear regression model.

Mean ± SEM (µg/mL)		
Timepoint	EC_10_	EC_50_
48 h	24.48 ± 0.53	220.33 ± 4.82
72 h	23.83 ± 0.14	214.47 ± 1.30

**Table 2:** Primer sequences used for real-time PCR analysis.

Gene	Forward Primer (5′–3′)	Reverse Primer (5′–3′)	Accession Number
*β-Actin*	GCGCGGCTACAGCTTCA	CTTAATGTCACGCCACGATTTCC	NM_001101.5
*CAT*	ACATCTGAAGGATCCGGACA	ATGCAGAGACTCAGGACGTA	NM_001752.4
*GPX1*	GGCAAGGAGAAACGCCCAAGA	AGCATGAAGTTGGGCTCGAA	NM_001329455.2
*GSR*	GAGGTGCTGAAGTTCTCCCA	TGACTTCCAAGCCCGACAAA	NM_000637.5
*GSTP1*	TGGTGGACATGGTGAATGAC	AGATGTATTTGCAGCGGAGG	NM_000852.4
*HMOX1*	CAGTCAGGCAGAGGGTGATA	CTCCTCAAAGAGCTGGATGTT	NM_002133.3
*Keap1*	CAGATTGGCTGTGTGGAGTT	TTGGCCACCTCCCCAAAAT	NM_203500
*NFE2L2*	CAGCTTTTGGCGCAGACATT	AAGTGACTGAAACGTAGCCGA	NM_006164.5
*NQO1*	CCAGAAAGGACATCACAGGTAA	AGACTCGGCAGGATACTGAA	NM_001025434.2
*SOD1*	GAGACCTGGGCAATGTGACT	GTTTACTGCGCAATCCCAAT	NM_000454.5

## Data Availability

The original contributions presented in this study are included in the article. Further inquiries can be directed to the corresponding author.

## Abbreviations

The following abbreviations are used in this manuscript:

CAT	Catalase
DCF-DA	2′,7′-Dichlorofluorescin Diacetate
DEGs	Differentially Expressed Genes
DMEM	Dulbecco’s Modified Eagle’s Medium
EC_10_/EC_50_	Effective Concentration 10%/50%
ECM	Extracellular Matrix
FBS	Fetal Bovine Serum
FC	Fold Change
FDR	False Discovery Rate
GPCR	G-Protein-Coupled Receptor
GPX	Glutathione Peroxidase
GSR	Glutathione Reductase
HaCaT	Human Adult Low-Calcium Temperature Keratinocytes
H_2_DCFDA	2′,7′-Dichlorodihydrofluorescein Diacetate
H_2_O_2_	Hydrogen Peroxide
HEE	*Haberlea Rhodopensis* Ethanolic Extract
HMOX1	Heme Oxygenase-1
KE	Keratinocytes
KEAP1	Kelch-Like ECH-Associated Protein 1
KEGG	Kyoto Encyclopedia of Genes and Genomes
NQO1	NAD(P)H Quinone Dehydrogenase 1
NRF2/NFE2L2	Nuclear Factor Erythroid 2-Related Factor 2
PBS	Phosphate-Buffered Saline
PPI	Protein–Protein Interaction
RNA-seq	RNA Sequencing
ROS	Reactive Oxygen Species
RT-PCR	Real-Time Polymerase Chain Reaction
SD	Standard Deviation
SOD1	Superoxide Dismutase 1
SRB	Sulforhodamine B
TCA	Trichloroacetic Acid
